# Weight perceptions in a population sample of English adolescents: cause for celebration or concern?

**DOI:** 10.1038/ijo.2015.126

**Published:** 2015-08-04

**Authors:** S E Jackson, F Johnson, H Croker, J Wardle

**Affiliations:** 1 Health Behaviour Research Centre, Department of Epidemiology and Public Health, University College London, London, UK

## Abstract

**Objectives:**

To examine the proportion of normal-weight adolescents who consider themselves to be too heavy (size overestimation), and the proportion of overweight or obese adolescents who consider themselves to be about the right weight or too light (size underestimation), in large population-based samples collected over 8 years in England.

**Methods:**

Data were from the Health Survey for England between 2005 and 2012: an annual survey of households representative of the English population. We analysed data from 4979 adolescents (2668 boys, 2311 girls) aged 13 to 15 years old whose weight status was defined as normal weight or overweight/obese based on body mass index standard deviation scores (BMI-SDS) derived from objective measurements of height and weight and using International Obesity Task Force standards. Weight perception was based on the adolescent's choice from the following descriptors: ‘about the right weight', ‘too heavy' or ‘too light'.

**Results:**

The majority of normal-weight adolescents (83% of boys, 84% of girls) correctly identified themselves as ‘about the right weight'. Overestimation was uncommon, with only 7% of normal-weight teens (4% of boys, 11% of girls) identifying themselves as ‘too heavy'. In contrast, only 60% of overweight/obese adolescents (53% of boys, 68% of girls) correctly identified themselves as ‘too heavy', whereas 39% (47% of boys, 32% of girls) underestimated, identifying themselves as ‘about the right weight' or ‘too light'. There were no significant changes in BMI-SDS or body size estimation over time (2005–2012).

**Conclusions:**

Overestimation of body weight among normal-weight adolescents is relatively uncommon; potentially a cause for celebration. However, almost half of boys and a third of girls with a BMI placing them in the overweight or obese BMI range perceived themselves to be about the right weight. Lack of awareness of excess weight among overweight and obese adolescents could be a cause for concern.

## Introduction

Weight is a topic of considerable interest in modern society, but messages are discordant. The mass media promote ideal standards for beauty that place great emphasis on slenderness in women and lean muscularity in men,^1, 2^ which may be unattainable for many people. Meanwhile, mass media coverage of obesity often uses images of severely obese individuals, which could give the impression that medical criteria for overweight and obesity require exceptionally high body weights. Public health campaigns highlight the importance of healthy weight control, encouraging people to follow a balanced diet and take regular physical activity.^3, 4^ Meanwhile, grassroots movements campaign for size acceptance, and billion-dollar industries market diet plans and products promising rapid weight loss.

Given these conflicting messages, it is not surprising that adolescents can develop inaccurate perceptions of the extent to which their own weights deviate from recommended weights. In the literature on adolescent body image, there has been longstanding emphasis on the tendency of clinically normal-weight teens, particularly girls, to perceive themselves as overweight; often seen as motivating unnecessary weight control efforts, and in extreme cases, leading to eating disorders.^5, 6, 7^ However, there is also a risk that clinically overweight or obese adolescents may fail to recognise that they carry excess weight, undermining their appreciation of the need for healthier diet and activity behaviours.^8, 9, 10, 11^

A number of studies have examined rates of under- and overestimation of body size in adolescent samples, with results indicating that up to 25% of normal-weight girls and around 5% of normal-weight boys perceive themselves to be too heavy.^6, 9, 12, 13, 14, 15^ There is also evidence that up to 48% of overweight girls and up to 61% of overweight boys perceive themselves to be about the right weight.^9, 10, 12, 13, 14, 15^ However, most of these studies are based on data collected in the United States of America in the 1990s and 2000s, and cannot be assumed to reflect current weight perceptions or to generalise to other populations. It is possible that rising levels of adiposity across the population have ‘normalised' overweight and obesity, leading to reduced recognition of excess weight,^16, 17^ a change that might make adolescents across all weight categories less likely to perceive themselves to be overweight. On the other hand, increased media and public health focus on obesity might have improved the recognition of excess weight among those who are overweight/obese and reduced concern about overweight in those who are normal weight.^4, 18^ Studies in adults have shown a decline in recognition of overweight over the past couple of decades,^16, 17, 19^ highlighting the need for contemporary data in adolescents.

The aim of the present study was therefore to investigate weight perceptions in large, nationally representative population samples of adolescents living in England collected over an 8-year period. Specifically, we examined the proportion of normal-weight adolescents who considered themselves to be overweight (size overestimation), and the proportion of overweight and obese adolescents who considered themselves to be a healthy weight (size underestimation), comparing effects by gender and survey year.

## Subjects and Methods

### Study population

We used data from 13 to 15 year olds collected as part of the Health Survey for England (HSE). Detailed information about the Health Survey for England's methodology has been published elsewhere.^20^ Briefly, it is an annual survey that uses a multistage, stratified, probability design to identify nationally representative samples of the general population of England. Participants are all adults, and up to two children (age <16 years; selected at random in families with three or more eligible children), in participating households. Heights and weights are measured by trained researchers in the participants' homes. Participants aged 8 years and older also fill in age-appropriate self-completion questionnaire booklets.

Since 2005, the self-completion booklet for 13–15 year olds has included a question on weight perception (this was not included in the booklets for other age groups). Across all survey years from 2005 to 2012, a total of 5850 13–15 year olds completed the booklet. For the present analyses, we excluded 623 participants with missing data on height, weight or key demographic variables. We also excluded underweight participants because numbers were too small for meaningful analysis (*n*=248), leaving a final analytic sample of 4979 (2668 boys and 2311 girls).

This study was carried out under ethical exemption from the University College London Ethics Committee because it used anonymous data in the public domain (Office for National Statistics, London, UK), where appropriate permission was already obtained. Ethical approval for the original data collection was granted by the London Multicentre Research Ethics Committee.

### Measures

Height was measured with a portable stadiometer (Chasmors Ltd, London, UK) and weight with electronic digital scales (Tanita Inc., Tokyo, Japan). body mass index (BMI) was calculated as the square of weight (kg) per height (m), and values were converted to standardised scores (BMI-SDS) and centiles relative to 1990 UK reference data using the Excel LMS Growth macro.^21, 22^ Weight status was defined according to the International Obesity Taskforce criteria,^23^ which classify BMI values according to age and sex as thin (underweight), normal weight, overweight or obese, based on adult BMI cutoffs at 18 years. For some analyses, we grouped together overweight and obese participants. For some analyses, normal-weight participants were divided into those with a BMI-SDS ⩽50th centile (termed ‘lower normal weight') and BMI-SDS >50th centile (‘upper normal weight').

Weight perception was assessed with the question 'Given your age and height, would you say that you are about the right weight, too heavy or too light'.

Demographic information included in the analyses were age, sex, ethnicity and socioeconomic status (SES). Ethnicity was self-reported by the adolescent and categorised as white (white British, white Irish, other white ethnic group), black (black African, black Caribbean, other black ethnic group), Asian (Indian, Pakistani, Bangladeshi, Chinese, other Asian ethnic group) or other (Arab, mixed/multiracial ethnic group, any other ethnic group). SES was indexed on the basis of the occupation of the household reference person (defined as the householder with the highest income) using the National Statistics Socio-Economic Classification.^24^ For the present analyses, we generated three categories: managerial and professional occupations (higher SES), intermediate occupations (intermediate SES) and routine and manual occupations (lower SES).

### Statistical analysis

All analyses were conducted in SPSS version 20, with a *P-*value <0.05 indicating statistical significance. To produce representative estimates for adolescents in the English population, data were weighted to account for differential probabilities of selection and participation based on age group, sex, government office region, household type and the social class of the household reference person. We pooled data from all survey years but included survey year as a variable to examine time trends. We conducted analyses separately for boys and girls. Sex differences were tested using *t*-tests (continuous variables) and *χ*^2^ analyses (categorical variables), and differences across survey years were tested using one-way independent analyses of variance (continuous variables) and *χ*^2^ analyses (categorical variables). We used multivariable logistic regression to identify unique predictors of overestimation (perception of weight as ‘too heavy') among normal-weight respondents, and underestimation (perception of weight as ‘about right' or ‘too light') among overweight/obese respondents. Variables in the models were age, ethnicity, SES, BMI (lower vs upper normal weight in normal-weight respondents; overweight vs obese in overweight/obese respondents) and survey year. There was no multicollinearity present in the models. We tested for changes in BMI-SDS across surveys using multiple linear regression, adjusting for age, ethnicity and SES.

## Results

Table 1 presents descriptive data overall and by sex. The analysed sample comprised 2668 boys and 2311 girls aged between 13 and 15 years (mean 14.0 years). The ethnic breakdown of the sample closely resembled England's 2011 census results for the whole population,^25^ with 86% of adolescents self-identifying as white, 7% as Asian, 3% as black and 4% as other (including mixed ethnic groups). Participants were distributed across socioeconomic groups (44% higher SES, 22% intermediate, 35% lower SES). The majority (73%) of adolescents had a BMI placing them in the normal-weight range, but 20% were overweight and 7% were obese. Boys were on average slightly taller and heavier than girls (*P*s <0.001), but there was no significant sex difference in BMI-SDS, weight status or any demographic variable.

### Weight perception among normal-weight adolescents

**Figure 1:**
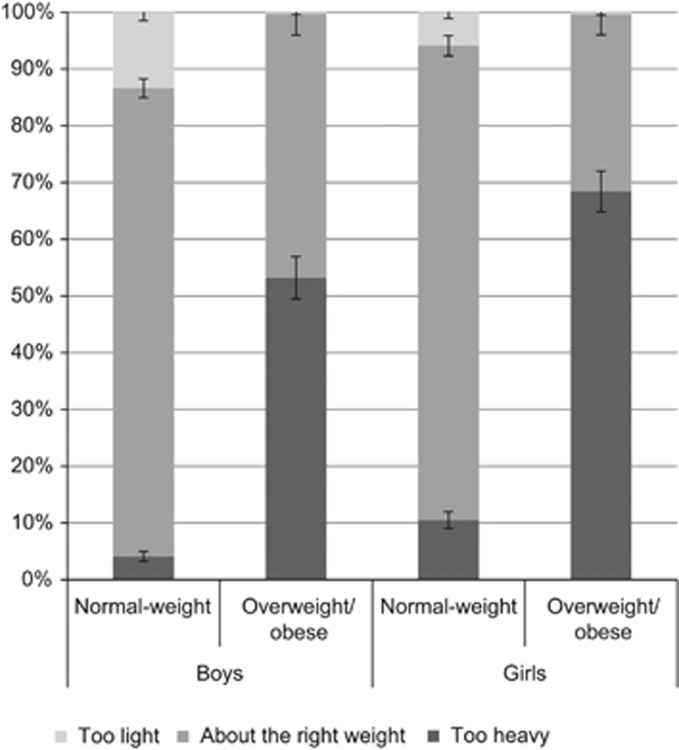
The proportion (with 95% CI) of boys and girls who reported feeling ‘too heavy', ‘about the right weight' and ‘too light', by measured weight status. Weighted data are shown.

Figure 1 shows weight perceptions by sex and weight status. In the normal-weight category, 83% of adolescents correctly identified themselves as about the right weight, but 7% thought they were too heavy, and 10% thought they were too light. Normal-weight girls were significantly more likely compared with normal-weight boys to consider themselves to be too heavy (11% vs 4%, *P*<0.001) and less likely to consider themselves too light (6% vs 13%, *P*<0.001).

Multivariable models examining factors independently associated with overestimation of body weight in normal-weight boys and girls (self-identification as ‘too heavy') are shown in Table 2. Overestimation was more likely among those in the upper normal-weight group (10%) compared with that in the lower normal-weight group (2%) with significant effects in both sexes (boys: odds ratio (OR)=20.85, *P*=0.007; girls: OR=3.65, *P*<0.001). There were no significant independent associations with age, ethnicity or SES, in either sex.

### Weight perception among overweight/obese adolescents

In adolescents whose weight placed them in the overweight/obese category, 60% felt they were too heavy, but 39% thought they were about the right weight, and 0.4% felt they were too light. Overweight/obese girls were more likely compared with boys to recognise that they were too heavy (68% vs 53%, *P*<0.001) and less likely to perceive themselves to be about the right weight or too light (32% vs 47%, *P*<0.001) (Figure 1).

Factors independently associated with underestimation of body weight in multivariable analyses in overweight/obese boys and girls are shown in Table 3. There was a strong association with weight status, with those in the overweight category much more likely to underestimate (52%) compared with those who were obese (7%). There was no consistent effect of ethnicity or SES on underestimation, but the odds of underestimation declined significantly with age in girls (*p*=0.039).

### Time trends in overestimation and underestimation of body weight: 2005–2012

BMI-SDS did not differ significantly across survey years in either boys (*P*=0.875) or girls (*P*=0.452). To test whether overestimation had decreased among normal-weight adolescents or underestimation had increased among overweight/obese adolescents between 2005 and 2012, survey year was included as a continuous independent variable in the models summarised in Tables 2 and 3. Although there was variation in prevalence between survey years, there was no significant time trend in overestimation among normal-weight boys (*P*=0.396) or girls (*P*=0.885) nor with underestimation among overweight/obese boys (*P*=0.851) or girls (*P*=0.697) after adjusting for age, ethnicity, SES and BMI.

## Discussion

This study examined the proportion of normal-weight adolescents who consider themselves to be too heavy (size overestimation), and the proportion of overweight or obese adolescents who consider themselves to be about the right weight (size underestimation), in a large population-based sample of 13–15 year olds. Our results showed relatively low levels of overestimation among normal-weight adolescents, with just 4% of boys and 11% of girls perceiving themselves to be ‘too heavy'. However, size underestimation among adolescents with a BMI in the overweight/obese range appeared to be much more prevalent, with almost half (47%) of boys and a third (32%) of girls perceiving themselves to be ‘about the right weight' or ‘too light'. These findings are consistent with results of several previous studies of adolescent weight perceptions, indicating higher rates of underestimation among overweight and obese teens compared with overestimation among those who are normal weight.^9, 12, 14, 15^

The finding that overestimation was more common among normal-weight girls compared with boys is also in line with previous findings^12, 13, 14, 15^ and likely reflects greater societal pressure towards, and expectations for, thinness in women.^26, 27, 28, 29^ Underestimation was also significantly lower in overweight/obese girls compared with overweight/obese boys, and the sex difference for underestimation was much larger compared with that for overestimation, suggesting that overall, girls had a more accurate recognition of weight status.

In addition to producing general estimates by pooling data across the eight surveys, we also examined whether underestimation and overestimation had changed substantially over time. Results showed no significant effect of survey year in either sex, providing no evidence for notable change in the prevalence of underestimation among overweight/obese adolescents or overestimation among normal-weight adolescents between 2005 and 2012. The latter finding mirrors the observed lack of significant change in weight perceptions over a similar time period (2007–2012) in obese adult men in England in a recent study.^30^

It is encouraging that rates of size overestimation among normal-weight adolescents, particularly girls, were relatively low, despite continued emphasis in popular western culture on achieving and maintaining a slender, toned physique. Nonetheless, there may be room to improve recognition of healthy body weight among adolescent girls, particularly those in the upper normal-weight range, where the prevalence of overestimation was higher (14% vs 4% in lower normal-weight range). Previous studies have indicated that perceived overweight in normal-weight adolescents can lead to unnecessary, sometimes unhealthy, dieting behaviours,^5, 6, 7^ and in extreme cases may result in clinical eating disorders.^31^ In the context of today's public health focus on curbing the incidence of obesity, there may be a temptation to focus efforts on strategies for reducing weight among overweight adolescents. It is important that obesity prevention programmes targeted at this age group also include educational components that address the risks of unhealthy weight control behaviours, regardless of BMI status.

The relatively high prevalence of size underestimation among overweight and obese adolescents may have implications for the future health and well-being of young people. Overweight and obesity have been shown to track from adolescence into adulthood, with overweight teens at substantially raised risk of becoming overweight adults.^32^ Previous studies have demonstrated strong associations between accurate weight perception and efforts to control weight in adolescents,^8, 9, 10^ suggesting that those who fail to recognise their weight status may be at greater risk of continued weight gain and obesity-associated comorbidities in later life compared with those who recognise they are overweight. This presents some challenging questions: whether and how to promote accurate weight perceptions and whether to proactively promote weight control efforts—for example, would weight loss or prevention of weight gain be most appropriate for this age group?

Raising awareness of weight status is challenging. Reliance on visual estimation is inaccurate as demonstrated in this study and others.^9, 10, 12, 13, 14, 15^ Opportunistic identification aside, a universal screening programme would seem the only viable method of raising weight awareness objectively at a population level. These have been used most widely used in children of primary school age (4–11 years), although to date success has been largely limited to improving parental awareness of weight status,^33, 34, 35^ with behaviour change more elusive.^33^ This approach also presents considerable logistical and financial challenges.

In relation to promoting weight control, studies have shown that many adolescents engage in healthy diet and physical activity practices, but the proportion engaging in unhealthy and even harmful weight control practices is unclear.^9^ It is important that efforts to promote a healthy weight in adolescents are mindful of potential harm. Structured obesity management programmes^36^ and obesity prevention programmes^37^ are not typically detrimental for adolescents' psychological functioning. The impact of unstructured weight loss efforts is less clear, but appears to be associated with less successful weight management and a greater risk of disordered eating.^38^ Although the integral role of parents in child weight management is well accepted,^39^ parents are also an important influence on adolescent behaviour, and it is generally recommended that they are involved to some extent in treatment.^40^ As well as acting as role models and influencing food availability in the home, parents can have an influence through the messages they portray about weight. In particular, parental engagement in conversations around healthy eating rather than dieting or weight appears to lessen the risk of adolescents engaging in unhealthy dieting behaviours.^41, 42^ Simple strategies such as these could be promoted to parents to encourage healthy behaviours and appropriate weight control efforts in their adolescent offspring. An alternative approach could be school-based initiatives, which have had some success in terms of weight and behavioural outcomes in adolescents.^43, 44^

This study benefited from a large, population-based sample of adolescents living in England, with analyses weighted to match key population characteristics. Objective measures of height and weight were an advantage given previous data showing that adolescents tend to overestimate their height, and particularly among those who are overweight/obese, to underestimate their weight.^45, 46^ Data across eight consecutive years allowed us to assess trends over time. However, there were also limitations. Weight measurements were not available for all adolescents included in the Health Survey for England (11% missing). If those who were more concerned about their weight were more likely to decline to be measured, our results may slightly underestimate the proportion of normal-weight adolescents who consider themselves to be too heavy, and overestimate the proportion of overweight/obese adolescents who consider themselves too heavy. It is very difficult to tell whether this was the case, as this survey is currently the best source of information on adolescent weight status in England. Items on weight perceptions were only included in questionnaires administered to 13–15 year olds, limiting the scope to examine variation across different periods of adolescence.

The relatively low prevalence of overestimation of body weight among normal-weight adolescents is potentially a cause for celebration given the longstanding concerns regarding unnecessary body dissatisfaction in adolescent girls. However, almost half of boys and a third of girls with a BMI placing them in the overweight or obese range perceived themselves to be about the right weight. Lack of awareness of excess weight among overweight and obese adolescents could be a cause for concern. Our findings highlight a disparity between the enduring focus of adolescent weight perceptions research—size overestimation in normal-weight teens—and the more prevalent issue of size underestimation among overweight teens. There is a need to develop methods of improving recognition of overweight and obesity among adolescents that do not cause unnecessary concern over weight in those whose weight is healthy.

## Figures and Tables

**Table 1:** Demographic and anthropometric characteristics of 13–15 year olds in the Health Survey for England 2005–2012^a^

	*Whole sample (*n=*4979)*	*Boys (*n=*2668)*	*Girls (*n=*2311)*	P*-value*^b^
Age (years)	14.01 (0.82)	14.04 (0.81)	13.98 (0.82)	0.093

*Ethnicity*				
White	85.5	85.4	85.6	0.652
Black	3.4	3.5	3.3	—
Asian	7.3	7.7	6.9	—
Other	3.8	3.4	4.2	—

*SES*				
Higher	43.9	45.1	42.6	0.513
Intermediate	21.5	21.2	21.9	—
Lower	34.6	33.7	35.5	—
Height (cm)	164.95 (8.89)	167.91 (9.49)	161.52 (6.67)	<0.001
Weight (kg)	59.31 (12.85)	60.80 (13.85)	57.59 (11.36)	<0.001
BMI-SDS	0.77 (1.03)	0.80 (1.05)	0.73 (1.01)	0.128
BMI centile	69.35 (25.58)	69.97 (25.70)	68.63 (25.44)	0.230

*Weight status*				
Normal-weight	72.8	73.0	72.7	0.174
Lower normal weight	25.8	25.0	26.8	—
Upper normal weight	47.0	48.0	45.9	—
Overweight	19.7	18.7	20.8	—
Obese	7.5	8.3	6.5	—

Abbreviations: BMI-SDS, body mass index standard deviation score; s.d., standard deviation; SES, socioeconomic status.

Weighted means and proportions are shown. Sample sizes (*n*) are shown unweighted.

a Overall and by sex, mean (s.d.) or %.

b *P*-values are for the association between each variable and sex.

**Table 2:** Predictors of size overestimation (feeling ‘too heavy') among normal-weight adolescents (multivariable analysis)

	*Boys (*n=*1982)*			*Girls (*n=*1667)*		
	*%*^a^	*OR (95% CI)*	P*-value*	*%*^a^	*OR (95% CI)*	P*-value*
Age (years)^b^	—	0.71 (0.46–1.11)	0.134	—	1.05 (0.78–1.40)	0.770

*Ethnicity*						
White	4.0	1.00	—	11.1	1.00	—
Black	7.4	2.01 (0.43–9.41)	0.377	15.0	1.44 (0.36–5.77)	0.611
Asian	5.9	1.41 (0.44–4.54)	0.563	3.3	0.34 (0.07–1.60)	0.171
Other	0	—	0.998	7.7	0.71 (0.17–3.01)	0.638

*SES*						
Higher	5.6	1.00	—	10.3	1.00	—
Intermediate	2.7	0.50 (0.18–1.40)	0.184	9.5	0.92 (0.47–1.78)	0.795
Lower	2.7	0.50 (0.21–1.20)	0.120	11.3	1.14 (0.66–1.97)	0.628

*Weight status*						
Lower normal weight	0.3	1.00	—	4.2	1.00	—
Upper normal weight	6.1	20.85 (2.32–187.43)	0.007	14.2	3.65 (1.88–7.07)	<0.001
						
*Survey year*^b^	—	1.07 (0.92–1.25)	0.396	—	1.01 (0.90–1.13)	0.885
2005	3.8	—	—	13.2	—	—
2006	1.4	—	—	8.5	—	—
2007	9.0	—	—	14.2	—	—
2008	4.5	—	—	7.8	—	—
2009	1.5	—	—	13.9	—	—
2010	5.7	—	—	13.3	—	—
2011	4.2	—	—	10.3	—	—
2012	4.2	—	—	9.3	—	—

Abbreviations: CI, confidence interval; OR, odds ratio; SES, socioeconomic status.

Weighted data. Sample sizes (*n*) are shown unweighted.

a The percentage of normal-weight participants in each group perceiving themselves to be too heavy.

b Entered into the model as a continuous variable. OR indicates the odds of size overestimation associated with each 1-year increase in age or survey year.

**Table 3:** Predictors of size underestimation (feeling ‘about the right weight' or ‘too light') among overweight/obese adolescents (multivariable analysis)

	*Boys (n=686)*			*Girls (n=644)*		
	*%*^a^	*OR (95% CI)*	P*-value*	*%*^a^	*OR (95% CI)*	P*-value*
Age (years)^b^	—	1.01 (0.73–1.40)	0.969	—	0.68 (0.47–0.98)	0.039

*Ethnicity*						
White	45.8	1.00	—	29.4	1.00	—
Black	56.4	1.80 (0.47–6.80)	0.389	29.1	1.67 (0.43–6.48)	0.456
Asian	37.0	1.15 (0.42–3.17)	0.787	58.5	3.12 (0.99–9.85)	0.052
Other	74.6	3.44 (0.84–14.02)	0.085	39.8	1.91 (0.56–6.48)	0.299

*SES*						
Higher	57.1	1.00	—	40.1	1.00	—
Intermediate	39.1	0.52 (0.25–1.05)	0.069	23.1	0.50 (0.23–1.07)	0.073
Lower	42.2	0.57 (0.31–1.05)	0.070	28.1	0.59 (0.31–1.12)	0.107

*Weight status*						
Obese	8.5	1.00	—	5.0	1.00	—
Overweight	63.9	19.22 (8.69–42.51)	<.001	39.9	12.79 (3.86–42.42)	<0.001

*Survey year*^b^	—	0.99 (0.88–1.12)	0.851	—	0.98 (0.86–1.11)	0.697
2005	36.3	—	—	28.2	—	—
2006	46.3	—	—	36.3	—	—
2007	46.1	—	—	25.9	—	—
2008	43.7	—	—	33.2	—	—
2009	41.1	—	—	44.5	—	—
2010	59.0	—	—	15.3	—	—
2011	57.0	—	—	33.9	—	—
2012	39.9	—	—	33.1	—	—

Abbreviations: CI, confidence interval; OR, odds ratio; SES, socioeconomic status.

Weighted data. Sample sizes (*n*) are shown unweighted.

a The percentage of overweight participants in each group perceiving themselves to be about the right weight.

b Entered into the model as a continuous variable. The OR indicates the odds of size underestimation associated with each 1-year increase in age or survey year.
